# Characteristics and Resources of Social Support Among Individuals in Recovery from Substance Use Disorders in Therapeutic Communities

**DOI:** 10.1192/j.eurpsy.2026.10504

**Published:** 2026-07-31

**Authors:** R. A. Bosso, M. A. D. Santos, A. Diehl, A. R. D. Almeida, A. L. Granjeiro, J. D. O. Bertholini, V. D. Santos, S. C. Pillon

**Affiliations:** 1 Psiquiatria; 2 Psicologia, Universidade de São Paulo - USP, Ribeirão Preto; 3 Psiquiatria, ABEAD, Londrina; 4 Psiquiatria, Unicamp; 5 Psicologia, Pesquisador Autônomo, Campinas, Brazil; 6 Psicologia, Fundação Universitária Iberoamericana - FUNIBER, Barcelona, Spain; 7 Psicologia, Faculdade Anhanguera, Campinas, Brazil

## Abstract

**Introduction:**

Social support is a recognized protective factor in the recovery of individuals with substance use disorders (SUDs), contributing to relapse reduction and treatment retention. Sociodemographic conditions can either enhance or restrict access to emotional, instrumental, and community support networks, especially in vulnerable settings such as therapeutic communities (TCs) in Brazil.

**Objectives:**

To analyze the sociodemographic profiles and perceived social support of individuals undergoing treatment for SUDs, and to discuss how these conditions influence access to different forms of social support over the recovery process.

**Methods:**

This is a cross-sectional, descriptive study with 307 participants who use psychoactive substances, admitted in two TCs in the Southeast region of Brazil. The following instruments were applied: sociodemographic questionnaire; the Family/Social Status domain (domain 6) of the Addiction Severity Index (ASI); the Spirituality Self-Rating Scale (SSRS); and the MOS Social Support Survey (MOS-SSS).

**Results:**

The sample was predominantly male (83.4%), non-white (52.8%), single (65.9%), and with children (67.6%, of whom 45.1% had minors). Religious affiliation was high (83.7%); Catholic (41.2%) and Evangelical (38.6%) predominated; 60.9% reported regular religious practice. Although 80.5% had worked in the last year, 44.0% received government benefits. Social vulnerability was significant: 37.0% had a history of homelessness, and 44.4% had no monthly income. Regarding social support, most participants reported receiving some support, especially emotional and religious, even amid limitations in instrumental support and family networks. Participants with weakened family ties tended to rely more on institutional support provided by TCs and public social assistance. Those who maintained frequent contact with family and had prior employment history reported greater perceived instrumental and motivational support, while religious ties emerged as an important source of affective and spiritual support during treatment. This set of conditions indicates fragility in family support, associated with unstable partnerships and economic hardship, but also reveals protective elements such as religious involvement and past work experience, which may facilitate access to emotional and instrumental support.

**Conclusions:**

The sociodemographic profile highlights vulnerabilities and protective resources that directly influence access to social support during recovery. We advocate that care strategies in TCs incorporate income generation and housing interventions, linked to strengthening family, religious, and peer ties, as a means to expand support networks and promote more protective treatment environments.

**Disclosure of Interest:**

None Declared

